# Routine saliva testing for SARS-CoV-2 in children: Methods for partnering with community childcare centers

**DOI:** 10.3389/fpubh.2023.1003158

**Published:** 2023-02-03

**Authors:** Erica J. Rayack, Hibah Mahwish Askari, Elissa Zirinsky, Sarah Lapidus, Hassan Sheikha, Chikondi Peno, Yasaman Kazemi, Devyn Yolda-Carr, Chen Liu, Nathan D. Grubaugh, Albert I. Ko, Anne L. Wyllie, Erica S. Spatz, Carlos R. Oliveira, Amy K. Bei

**Affiliations:** ^1^Yale School of Nursing, Orange, CT, United States; ^2^Department of Epidemiology of Microbial Diseases, Yale School of Public Health, New Haven, CT, United States; ^3^Department of Pediatric Infectious Diseases, Yale School of Medicine, New Haven, CT, United States; ^4^Department of Pathology, Yale School of Medicine, New Haven, CT, United States; ^5^Department of Cardiology, Yale School of Medicine, New Haven, CT, United States

**Keywords:** SARS-CoV-2, routine testing, childcare, COVID-19, SalivaDirect, saliva

## Abstract

While considerable attention was placed on SARS-CoV-2 testing and surveillance programs in the K-12 setting, younger age groups in childcare centers were largely overlooked. Childcare facilities are vital to communities, allowing parents/guardians to remain at work and providing safe environments for both children and staff. Therefore, early in the COVID-19 pandemic (October 2020), we established a PCR-based COVID-19 surveillance program in childcare facilities, testing children and staff with the goal of collecting actionable public health data and aiding communities in the progressive resumption of standard operations and ways of life. In this study we describe the development of a weekly saliva testing program and provide early results from our experience implementing this in childcare centers. We enrolled children (aged 6 months to 7 years) and staff at seven childcare facilities and trained participants in saliva collection using video chat technology. Weekly surveys were sent out to assess exposures, symptoms, and vaccination status changes. Participants submitted weekly saliva samples at school. Samples were transported to a partnering clinical laboratory or RT-PCR testing using SalivaDirect and results were uploaded to each participant's online patient portal within 24 h. SARS-CoV-2 screening and routine testing programs have focused less on the childcare population, resulting in knowledge gaps in this critical age group, especially as many are still ineligible for vaccination. SalivaDirect testing for SARS-CoV-2 provides a feasible method of asymptomatic screening and symptomatic testing for children and childcare center staff. Given the relative aversion to nasal swabs in younger age groups, an at-home saliva collection method provides an attractive alternative, especially as a routine surveillance tool. Results can be shared rapidly electronically through participants' private medical chart portals, and video chat technology allows for discussion and instruction between investigators and participants. This study fosters a cooperative partnership with participating childcare centers, parents/guardians, and staff with the goal of mitigating COVID-19 transmission in childcare centers. Age-related challenges in saliva collection can be overcome by working with parents/guardians to conceptualize new collection strategies and by offering parents/guardians continued virtual guidance and support.

## Introduction

As SARS-CoV-2 transmission continues, vaccination efforts proceed, and local outbreaks fluctuate, surveillance remains a valuable tool for mitigating the effects of COVID-19. Early in the pandemic, young children were ineligible for vaccine; and with the current low uptake of vaccines in children under 5 years of age in the United States, screening for SARS-CoV-2 stands as a vital control strategy, with ongoing research into the acceptability and effectiveness of screening methods remaining an integral component. While testing and surveillance efforts in the K-12 setting were frequent topics of public discourse, younger age groups attending childcare centers were largely omitted from these discussions (1). Childcare facilities are a crucial part of communities, providing safe environments for both children and staff, while also permitting parents/guardians to work with little disruption (2). COVID-19 screening programs are of particular importance in childcare centers as they could identify infections early, prevent outbreaks, and keep centers open, particularly in times of high community viral transmission.

Saliva based testing for SARS-CoV-2 has been successfully implemented in the US and around the world (3). The use of saliva samples for testing of SARS-CoV-2 infections in children may increase the feasibility of a surveillance system by reducing the invasiveness in testing. SalivaDirect was granted Emergency Use Authorization from the U.S. Food and Drug Administration in August 2020 and showed greater sensitivity for the detection of SARS-CoV-2 than nasopharyngeal sampling, with low rates of false-positive and invalid results (4). When compared with midturbinate swabs, SalivaDirect was shown to have greater sensitivity earlier on in infection (5). This simple, effective, and non-invasive testing method provides an alternative to methods that require expensive additives and costly cooling approaches, making it suitable for the needs of large-scale testing (6).

The goals of this project were to mitigate outbreaks in childcare centers and nursery schools, provide actionable public health data, and aid communities in the progressive resumption of standard operations and ways of life by identifying cases early and preventing transmission. Our strategy centered on establishing strong partnerships with families and childcare centers directors and staff to inform study design and execution. The following is a guide to establishing a surveillance program in childcare facilities using SARS-CoV-2 saliva-based RT-PCR testing for children and staff.

## Materials and equipment

Saliva collection tubes (conical tubes ranging in size from 5 to 50 mL, with tight-fitting lids)
Disposable plastic bulb transfer pipettes
Tube labels
Biohazard sample storage bags
Portable, waterproof, sealable coolers (Coleman).

## Methods

### Recruitment and enrollment

Children between the ages of 6 weeks to 7 years and childcare staff of any age were eligible for enrollment in this study. Childcare facilities were selected in an attempt to cover various demographics within New Haven, however, ultimately, seven sites were enrolled based on interest in our testing program. During the study design phase, we began by organizing virtual town halls and small-group discussions with parents/guardians and staff of participating childcare facilities using video conference tools. The study team described the purpose, goals, and proposed design of the study, and staff and parents/guardians were given an opportunity to voice concerns and suggestions. We discussed the timing of saliva collection, teaching/observation method for collection (by video), and notification and timing of test results. The study design was iteratively updated with considerations for both staff and parent suggestions (in addition to IRB and health and safety requirements), recognizing the importance of partnering with childcare centers and increasing the likelihood of stakeholder buy-in and compliance within each center. The goal was to make this process easy for families and staff of these centers in order to ultimately improve the coverage and timeliness of testing results. Consent documentation was emailed or dropped off at childcare facilities for parents/guardians and staff to pick up at their convenience. Virtual meetings were scheduled to review and sign the consent with participants, capture basic demographic information, and complete the initial saliva collection training with a study team member.

### Saliva sample collection

Participants and parents/guardians received weekly collection materials including empty saliva collection tubes, disposable plastic bulb transfer pipettes, tube labels, and biohazard bags. These supplies were available for participants to pick up in designated coolers outside of the childcare centers each week. Initial saliva collections were conducted using video teleconferencing tools, with study staff guiding participants. Participants were provided with conical collection tubes approved by the testing facility (ranging in size from 5 to 50 mL) and asked to provide 0.5–1 mL of saliva. Saliva collection techniques varied by age, as outlined below. Participants collected samples at home in the evening, refrigerated them overnight, and dropped them off the following morning in a designated cooler outside of each center. Table 1 provides detailed instructions for saliva collection by age groups.

**Table 1:** Instructions for saliva collection.

**For parents/guardians with infants and children <3 years old:** 1. Collect sample right before the baby is going to feed. 2. If the child is a toddler, provide him/her with a chew toy to prevent the child from biting down on the pipette. 3. Squeeze the bulb of the pipette and insert it into the lower part of the front of the mouth, under the tongue where saliva will pool when a child is sitting upright. Alternatively insert the pipette along the cheeks if the baby is on his/her side. 4. Release the bulb to collect saliva once the pipette is in the mouth. 5. Remove the pipette from mouth and squeeze the bulb again to empty the saliva into the tube. 6. Repeat until roughly 0.5-1 mL of saliva is in the tube.
**For parents/guardians with children >3 years old:** 1. Place the child's favorite food either in front of them or in their hands. We suggest something they cannot easily start eating (for example, an apple, a baby food pouch, or a banana). Alternatively, instruct child to think of his/her favorite food. This will increase saliva production. 2. Allow child about 30 seconds to pool saliva in the mouth. 3. Place conical tube along lower lip and instruct child to spit gently into the tube. 5. Repeat until roughly 0.5-1 mL of saliva is in the tube.

Suggested instructions for saliva collection are divided by age group (<3 years of age and >3 years of age). These strategies are illustrated visually in Supplementary Video 1.

Participants were considered fully trained in the collection after 2–3 virtually supervised sessions and, after reporting no difficulty, could perform the subsequent collections unsupervised. If parents/guardians or staff had challenges following this, additional sessions were scheduled. An open “drop-in” teleconference link with a waiting room was made available where participants could join one by one the evening before scheduled drop-offs should they require additional assistance or coaching. Each week on the night prior to sample collection day, participants received an email to remind them to pick up collection materials, collect saliva samples at home, and drop them off at their respective childcare facilities the following morning. Participants were instructed to label their tube and place it in the biohazard bag for storage overnight in their refrigerator (or collect it in the morning before school). Designated coolers were provided to the childcare facilities to facilitate the sample drop-off, and liaisons at the childcare facilities were reminded to place the coolers in the drop-off area for parents/guardians.

### Transportation, storage, and processing

Standard, household coolers containing ice packs were used for transporting samples. Samples were packaged as a sealed sample tube, inside a sealed biohazard bag with absorbent material, inside the sealed leakproof cooler bag. Samples which came back from the lab with an invalid—“leaked” error were contained within the biohazard bag. Following participant sample drop-off, coolers were collected by a designated study team member with approvals for transporting samples. They were transported to the Yale Pathology laboratory for clinical diagnostic testing, where they were processed under clinical laboratory improvement amendments (CLIA) regulations in a CLIA-approved laboratory within 24 h following Workflow One of the SalivaDirect RT-PCR assay (7), testing lysates for SARS-CoV-2 RNA on the Biorad CFX96 Touch or ThermoFisher 7500-DX (using the Bio-Rad SARS CoV-2 RT-PCR Master Mix). An additional measure that is used to monitor the success of sample collection is the detection of human RNase P (RP) in the SalivaDirect assay. This informs the laboratory if a sample was not of sufficient quality for testing. We received reports of “invalid” (insufficient RP signal) after each test to alert families that we could not give them a reliable test result but to also help to improve their sample collection to ensure a valid result the next time.

Participants were given access to the Yale-New Haven Hospital (YNHH) MyChart platform to view test results. Participants were not contacted in the event of a negative result unless specifically requested by the participant. Positive results were directly reported to the participant and reported to the study physician. The study physician then contacted the participant to provide guidance and instruction on isolation and continuity of care with the participant's primary care provider as needed. Parents/guardians and staff consented to notify their respective childcare facility in the case of a positive result. Childcare facility directors were also notified if no positives were detected that week, allowing for the continuation of routine operations.

### Data collection and analysis

Study data was collected and recorded using Research Electronic Data Capture REDCap electronic data capture tools hosted at Yale University (8, 9). Weekly surveys were also sent out using this platform. The survey allowed for the tracking of symptoms, recent travel and exposure histories, SARS-CoV-2 vaccination status, and the number of days of childcare attended per week. To ensure the reliability of screening, we monitored on an ongoing basis the trends and consistency of samples provided per participant, as well as the proportion of samples that did not pass quality control procedures. Positivity rates per center, cohort, and classroom were estimated on a weekly basis to ascertain if local circulation was occurring. To assess feasibility, we evaluated the participation rate and acceptability of study instruments.

## Results and lessons learned

**Figure 1:**
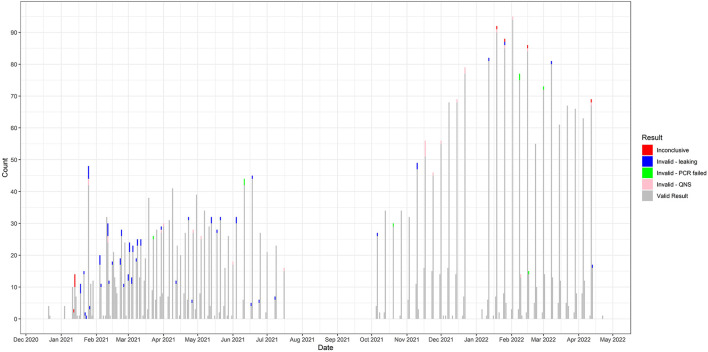
Testing results over time. Counts of test results over the course of the study are shown. Results include: inconclusive, invalid due to leaking, invalid due to PCR failure, invalid due to quantity not sufficient, and valid result.

From December 2020 until April 2022, 142 children and 124 childcare staff (266 total participants) agreed to participate in the study. A total of 3,509 saliva samples were collected during the study period. Testing numbers performed and the number of valid, invalid, and inconclusive tests are displayed in Figure 1. There were 3,405 valid tests, nine inconclusive, and 95 invalid SARS-CoV-2 test results. Of the 95 invalid, 61 were from leaking tubes, 26 were invalid-QNS (“Quantity Not Sufficient”), and eight were invalid due to a PCR failure. Family and community engagement was vital to the project's success and served as its foundation. Caregivers and childcare leadership were also considered partners and often met with research team members to provide meaningful suggestions on study protocols, help establish rapport with the childcare community, and provide access to useful local resources. The challenges encountered and potential remedies for implementing this surveillance system are summarized in Table 2. The anticipated overall results from this study will be as follows: (1) rates of asymptomatic SARS-CoV-2 positivity in children and teachers/staff, (2) incidence rates within classrooms of children/staff who test positive, (3) weekly test positivity rates, (4) a blueprint for testing strategies with a partnered, multi-stakeholder approach to testing and dissemination strategies, and (5) identification of key determinants of exposure risk in childcare settings.

**Table 2:** Challenges and potential remedies of weekly saliva testing in childcare centers. We identified the goals of the study and the testing program, the challenge encountered, and suggested potential remedies to maximize the utility of this routine testing program.

**Program goal**	**Challenge encountered**	**Potential remedies**
Maximal participation within each childcare center	Varying enrollment rates among centers	– Prior to beginning enrollment, host “Town Hall”-style video meetings for staff and parents/guardians – Use recruitment emails and flyers with quick response (QR) code for expedited enrollment requests
Weekly participation	Inconsistent weekly participation in saliva collection	– Send weekly reminder emails to all participants and childcare centers on the night of saliva collection, one day prior to collection date at the childcare facility. – Partner with a staff member at each center who acts as an on-site coordinator. This person monitors supply needs (e.g., tubes, biohazard bags, pipettes) and places cooler outside on collection days.
Sufficient sample quality and quantity	Difficulty with small children collecting enough saliva to run RT-PCR	– Train participants and parents on method for saliva collection using video chat technology for one-on-one instructional sessions – Provide age-adjusted collection materials (e.g., wider conical tubes, pipettes)
Efficient processing of saliva samples	Delays in transportation and processing of samples	– Urge participants to place saliva samples in designated coolers immediately upon arrival to center in the morning – Recruit designated courier to pick samples up and deliver to the lab in a timely manner – Communicate with laboratory regarding preferred days to process samples. This is especially important during periods of high community transmission when the laboratory is operating under high demand.
Timely reporting of results	Result turnaround period longer than 24 hours	– Have results go directly to patients via online patient portal – Arrange with laboratory to call or email study physician to report all positive results – Study physician calls all participants with positive results immediately

### Participation and acceptability

Age-related challenges in sample collection were overcome through engagement and discussion with parents/guardians and their children. In infants and younger toddlers, participating parents/guardians reported occasional difficulty using the pipette to retrieve the saliva sample. Guidance in the form of written instructions, visual aids, and optional weekly video chatting with study staff provided parents/guardians with the support needed to improve saliva collection feasibility for both infants and toddlers. In the older age groups, the width of a 5 mL conical tube was found to be suboptimal for saliva collection. We provided 50 mL conical tubes, which are notably wider and, when considering the coordination and dexterity of this age group, were found to be more appropriate. Frequent email communications sent to participants emphasized the availability of study staff to help with collection or attend to study-related concerns. Email reminders were sent the night before the day samples were to be collected. Notably, when reminder emails were not sent to parents/guardians and facility liaisons, participation for that specific week dropped dramatically, again elucidating the importance of frequent engagement between the study team and parents/guardians.

A considerable drop in inconclusive samples was seen as the study proceeded (Figure 1). Communication with the laboratory staff revealed insufficient volume or tube leakage as the main causes of inconclusive samples (Invalid-leaking, Invalid-QNS; Figure 1). Study materials were adjusted to increase the ease of tube closure, and reminder emails were sent to participants regarding the minimal saliva volume needed and the importance of tightening tube lids. The ability of participants to adapt to these instructions supports the feasibility of at-home saliva sample collection.

Despite the initial transient challenges to at-home saliva collection, it offers a less-invasive alternative to nasal/nasopharyngeal swabs in young children, especially when accompanied by written instruction for parents/guardians. Collection completion by parents/guardians and their children decreases the need for interaction with the healthcare workforce, thereby decreasing the risk of nosocomial infection and alleviating a major factor in testing bottlenecks (10, 11). It also alleviates the need for supplies, such as nasal swabs and personal protective equipment (10).

### Frequency of testing

The public health value of frequent asymptomatic SARS-CoV-2 testing has been emphasized in multiple analytic modeling studies (12, 13). Models have demonstrated that sporadic testing is likely to increase missed positive tests or first-time positive tests from individuals who have already passed their infectious period (7). Weekly testing was sufficient to provide effective attenuation of infection surges in models, while less frequent testing, or no testing, was not (12). Prompt reporting of results was also highly supported by these models (12). Based on the information presented in these models, weekly surveillance with next-day reporting of test results was implemented in this study.

Though more frequent testing is ideal for infection monitoring, our study was designed to balance effectiveness, feasibility, and the unique circumstances occurring at this time. For instance, Environmental Health and Safety protocols were frequently adjusted and updated in response to the changing state of the pandemic, resulting in our further reliance on tools, such as video communication and email discussion, as opposed to face-to-face approaches. Our methods can be practicably adapted to contexts beyond childcare facilities. Procedural adaptation must account for variations by context, including transmission dynamics, cost-effectiveness, and community features (12, 13).

## Conclusions

Childcare centers are critical to communities and provide services essential to society. Weekly screening for SARS-CoV-2 infections among children and staff may mitigate outbreaks and allow centers to remain open more consistently. Here, we conducted weekly screening using the SalivaDirect RT-PCR test. At-home saliva collection is a simple and non-invasive alternative to nasopharyngeal swabs and is optimally suited for routine and frequent testing for surveillance purposes outside a hospital setting. In partnership with childcare centers and parents/guardians, we were able to implement routine saliva-based testing for SARS-CoV-2 with ease in this setting. Weekly SalivaDirect testing in childcare centers will continue to prove beneficial as new variants continue to emerge and community rates fluctuate.

## Data availability statement

The original contributions presented in the study are included in the article and Supplementary material, further inquiries can be directed to the corresponding author.

## Ethics statement

The studies involving human participants were reviewed and approved by Human Investigation Committee of Yale University (Protocol 2000028639). Written informed consent to participate in the study was obtained from participants or participants' legal guardians/next of kin.

## Author contributions

AB, AK, ES, CO, and EZ were involved in the conception and design of the study. ER, HA, EZ, SL, HS, CP, YK, DY-C, CL, NG, AW, CO, and AB were involved in the implementation of the study protocol and the acquisition of data. All authors have been involved in the drafting of the manuscript and have approved the final submitted version.
